# Standardized Index of Shape (DCE-MRI) and Standardized Uptake Value (PET/CT): Two quantitative approaches to discriminate chemo-radiotherapy locally advanced rectal cancer responders under a functional profile

**DOI:** 10.18632/oncotarget.14106

**Published:** 2016-12-22

**Authors:** Antonella Petrillo, Roberta Fusco, Mario Petrillo, Vincenza Granata, Paolo Delrio, Francesco Bianco, Biagio Pecori, Gerardo Botti, Fabiana Tatangelo, Corradina Caracò, Luigi Aloj, Antonio Avallone, Secondo Lastoria

**Affiliations:** ^1^ Radiology Unit, Department of Diagnostic Imaging, Radiant and Metabolic Therapy, “Istituto Nazionale Tumori Fondazione Giovanni Pascale – IRCCS”, 80131, Naples, Italy; ^2^ Gastrointestinal Surgical Oncology Unit, Department of Abdominal Oncology, “Istituto Nazionale Tumori Fondazione Giovanni Pascale – IRCCS”, 80131, Naples, Italy; ^3^ Radiotherapy Unit, Department of Diagnostic Imaging, Radiant and Metabolic Therapy, “Istituto Nazionale Tumori Fondazione Giovanni Pascale – IRCCS”, 80131, Naples, Italy; ^4^ Scientific Director, “Istituto Nazionale Tumori Fondazione Giovanni Pascale – IRCCS”, 80131, Naples, Italy; ^5^ Diagnostic Pathology Unit, Department of Diagnostic and Laboratory Pathology “Istituto Nazionale Tumori Fondazione Giovanni Pascale – IRCCS”, 80131, Naples, Italy; ^6^ Nuclear Medicine Unit, Department of Diagnostic Imaging, Radiant and Metabolic Therapy, “Istituto Nazionale Tumori Fondazione Giovanni Pascale – IRCCS”, 80131, Naples, Italy; ^7^ Gastrointestinal Medical Oncology Unit, Department of Abdominal Oncology, “Istituto Nazionale Tumori Fondazione Giovanni Pascale – IRCCS”, 80131, Naples, Italy

**Keywords:** rectal cancer, neoadjuvant chemo-radiotherapy, DCE-MRI and FDG-PET/CT, treatment response assessment

## Abstract

**Purpose:**

To investigate dynamic contrast enhanced-MRI (DCE-MRI) in the preoperative chemo-radiotherapy (CRT) assessment for locally advanced rectal cancer (LARC) compared to^18^F-fluorodeoxyglucose positron emission tomography/computed tomography (^18^F-FDG PET/CT).

**Methods:**

75 consecutive patients with LARC were enrolled in a prospective study. DCE-MRI analysis was performed measuring SIS: linear combination of percentage change (Δ) of maximum signal difference (MSD) and wash-out slope (WOS). ^18^F-FDG PET/CT analysis was performed using SUV maximum (SUV_max_). Tumor regression grade (TRG) were estimated after surgery. Non-parametric tests, receiver operating characteristic were evaluated.

**Results:**

55 patients (TRG1-2) were classified as responders while 20 subjects as non responders. ΔSIS reached sensitivity of 93%, specificity of 80% and accuracy of 89% (cut-off 6%) to differentiate responders by non responders, sensitivity of 93%, specificity of 69% and accuracy of 79% (cut-off 30%) to identify pathological complete response (pCR). Therapy assessment via ΔSUV_max_ reached sensitivity of 67%, specificity of 75% and accuracy of 70% (cut-off 60%) to differentiate responders by non responders and sensitivity of 80%, specificity of 31% and accuracy of 51% (cut-off 44%) to identify pCR.

**Conclusions:**

CRT response assessment by DCE-MRI analysis shows a higher predictive ability than ^18^F-FDG PET/CT in LARC patients allowing to better discriminate significant and pCR.

## INTRODUCTION

Approximately forty thousand new cases of rectal cancer are accounting in the USA in 2015 [1]. Despite the introduction of the screening programs, several patients are diagnosed in a locally advanced stage. Preoperative radio-chemotherapy (pCRT) associated with total mesorectal excision (TME) is the standard care procedure for locally advanced rectal cancer (LARC) [2, 3]. TME is linked to morbidity and complications, therefore in clinical practise there is an increase of conservative treatment strategies application for patients with substantial tumor regression after pCRT and “wait and see” policy for patients with complete pathological regression. The advantage of this strategy is the reduction of morbidity and the possibility to provide a “true” organ-sparing approach. In this scenario is necessary to individuate the selection criteria for these strategies that accurately can assess neoadjuvant treatment response. Morphological MRI (mMRI) is the best tool for local LARC staging, permitting a correct assessment of the disease extent, of the mesorectal fascia and lymph node involvement [4, 5]. On the other hand, there are some limits to detect changes after pCRT by means of mMRI [4]. A positive tumor response may not correspond to a significant tumor size reduction. Moreover, it is difficult to discriminate between post treatment fibrosis and residual viable tumor using morphological approach. To overcome this limitation, functional approaches that aim to assess tissue “viability” through different imaging modalities such as Position Emission Tomography, Dynamic Contrast Enhanced-Magnetic Resonance Imaging (DCE-MRI), Diffusion Weighted Magnetic Resonance Imaging (DWI) are being actively investigated. One widely used approach is Positron Emission Tomography coupled with Computed Tomography (PET/CT) that in rectal cancer management is capable to early predict treatment response [6–10]. However, among data reported in literature [7–8, 10], late PET scans, performed before surgery, showed lower accuracy in pathologic response assessment.

Some authors described the value of mMRI and additional ^18^F-fluorodeoxyglucose positron emission tomography/computed tomography (^18^F-FDG PET/CT) for pCRT tumor response evaluation in patients with LARC [7, 8]. In Huh et al. [7] sensitivity, specificity and diagnostic accuracy of mMRI to predict pathologic complete response were 38.5%, 58.1% and 55.2%, respectively. Using a response index (percentage change of Standardized Uptake Value maximum, ΔSUVmax) of 63.6%, it was possible to detect the complete response response with a sensitivity of 73.1%, a specificity of 64.5% and an accuracy of 65.7%. Aiba et al. [8] have shown no benefit adding ^18^F-FDG PET/CT to mMRI in assessment of pCRT responders based on changes in area under receiver operating characteristic curve. To the best of our knowledge, there are no available studies in the literature on an enough number of patients that directly compare functional parameters obtained by^18^F-FDG PET and DCE-MRI in the pre-surgical evaluation of CRT in LARC. Using these imaging methods with the same timing allows exploring potential existing relationships between two different functional tissue proprieties: tumor vascularity investigated by tissue perfusion and tissue glucose metabolism [11–25].

In a previous study, we investigated a semi-quantitative analysis with DCE-MRI [14–20], finding the best combination, denominated Standardized Index of Shape (SIS), that identifies the linear classifier of the percentage differences Δ of Maximum Signal Difference (MSD) and of Wash-Out Slope (WOS) [7], with a sensitivity and specificity of 93.5% and 82.1% in discrimination of responder by non responder patients after pCRT [13].

The objective of this study was to validate the potential of SIS analysis in LARC to identify significant and pathological complete response after neoadjuvant preoperative CRT, in comparison with ^18^F-FDG PET.

## RESULTS

For both examinations, PET/CT and DCE-MRI, the temporal range between baseline and preoperative scan were 90 days (± 15). The median interval between the end of CRT and TME was 9 weeks for both regimen (range, 7–14). All patients in our series had a radical resection with an undamaged and complete mesorectum. Patient characteristics and clinical staging including Gunderson Risk [25], distance by anal verge, circumferential resection margin were reported in Table 1. 27 patients were classified as pT0 (pathological T), 7 as pT1, 31 as pT2, 9 as pT3, and 1 as pT4. There were 30 patients with a tumour regression grade (TRG) equal to 1, 25 with a TRG = 2, 10 with a TRG = 3, 10 with a TRG = 4, and none with a TRG = 5. Then, based on TRC classification 55 (73.3%) patients were classified as responders (TRG = 1-2) and 20 (26.7%) as non-responders (TRG = 3-4). No significant differences between pathological responders and non-responders could be found regarding patients characteristics (Table 1).

**Table 1:** Patient Characteristics and Clinical Staging

Characteristics	All patients n=75 (%)	TRG 1–2 n= 55	TRG 3–4 n=20	p*
*Gender*				>0.05
Male/Female	50(67)/25(33)	37/18	13/7	
Median age (range)	62(44-77)	62(44-77)	63(44-76)	
*Gunderson Risk (25)*				>0.05
Intermediate: T3N0	4(5.3)	3	1	
Moderately high: T3N1, T4N0	26 (34.7)	17	9	
High: T3N2, T4N1-2	45 (60.0)	33	12	
*Distance from the anal verge*				>0.05
≤ 5 cm	37(49)	28	9	
> 5 cm	38(51)	27	11	
*Circumferential resection margin*				>0.05
> 2 mm	32(43)	26	2	
>1 and ≤ 2 mm	9(12)	7	2	
≤ 1 mm	30(40)	20	10	
Not mesurable	4(5)	2	2	

* Chi-square test or Mann- Whitney U Test

Median values for ΔSIS and ΔSUV_max_ for responder and non-responder patients according to TRG (TRG 1-2 vs TRG 3-4) and pathological T (pT 0-2 vs pT3-4) are reported in Table 2. Mann-Whitney test showed statistically significant differences for ΔSIS and ΔSUV_max_ median values between responders and non-responders patient based on TRG. Statistically significant differences based on pT were only found for ΔSIS values (Table 2).

**Table 2:** ΔSIS and ΔSUV_max_ median values for patients subgroups depending on TRG and pT score

	All patients n=75	TRG 1–2 n= 55	TRG 3–4 n=20	p*
*ΔSUV_max_ median (standard deviation)*	62%(36)	73%(37)	48%(30)	< 0.001
*ΔSIS median (standard deviation)*	36%(65)	57%(49)	−18%(73)	< 0.001
		**pT 0–2 n= 65**	**pT 3–4 n=10**	p*
*ΔSUV_max_ median (standard deviation)*		66 (36)	58 (34)	>0.05
*ΔSIS median (standard deviation)*		42 (59)	−20 (80)	< 0.001

* Mann- Whitney U Test

**Figure 1:**
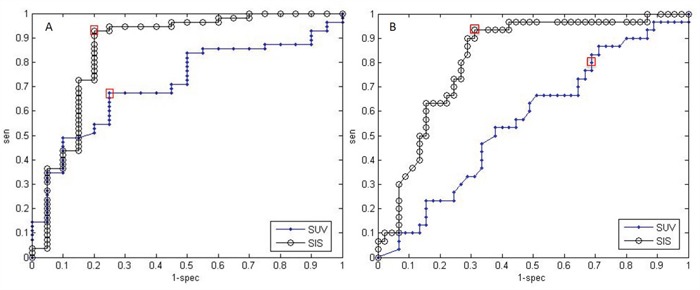
**A.** ROC analysis for ΔSIS and ΔSUVmax in discriminating responders from non responders. Cut-off value in ΔSIS (black line, AUC = 0.86) changed of 6.0% yields 92.7% of sensitivity and 80.0% of specificity. Cut-off value in ΔSUVmax (blue line, AUC = 0.71) of 59.7% gave a sensitivity of 67.3% and a specificity of 75.0%. **B.** ROC analysis for ΔSIS and ΔSUVmax to identify pathological complete response. Cut-off value in ΔSIS (black line, AUC = 0.82) changed of 30.3% yields 93.3% of sensitivity and 68.9% of specificity. Cut-off value in ΔSUVmax (blue line, AUC = 0.57) of 43.9% gave a sensitivity of 80.0% and a specificity of 31.1%. The two red rectangles highlight the sensitivities and specificities corresponding to the optimal thresholds.

**Figure 2:**
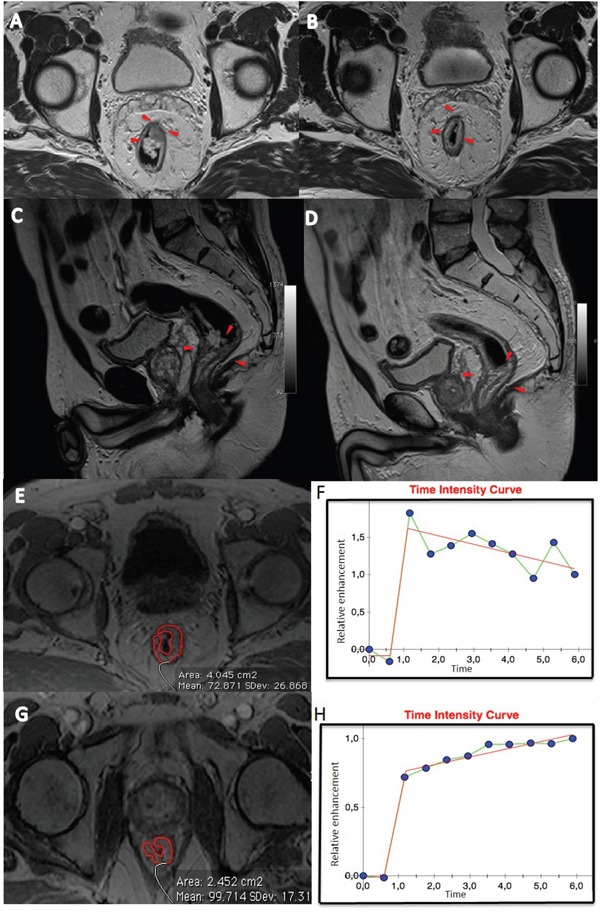
Patient n. 64: T2-weighted images in axial **A** and sagittal **C** plane before **B** and after treatment **D.** The morphologic images (A and C) before CRT, showed heterogeneous irregular thickening along the rectal wall spreading into the perirectal fat (A, arrowheads). After CRT, a hypointense area relating to rectal wall thickening is still visible (B and D, arrowheads). Median Time intensity curve of volume of interest (**E** and **G**), segmented by expert radiologist, before treatment is shown in **F** and after treatment in **H.** These curves showed different contrast enhancement, with a ΔSIS of 31.93% classifying the patient as responder.

**Figure 3:**
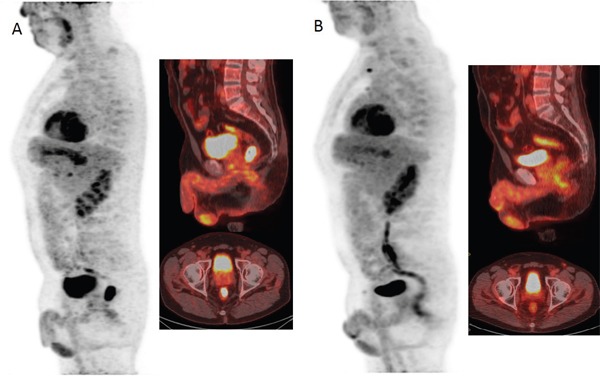
^18^F-FDG PET/CT images before A and after B treatment showed a reduction of FDG uptake with a ΔSUVmax of 96.67% classifying the patient as responder.

**Figure 4:**
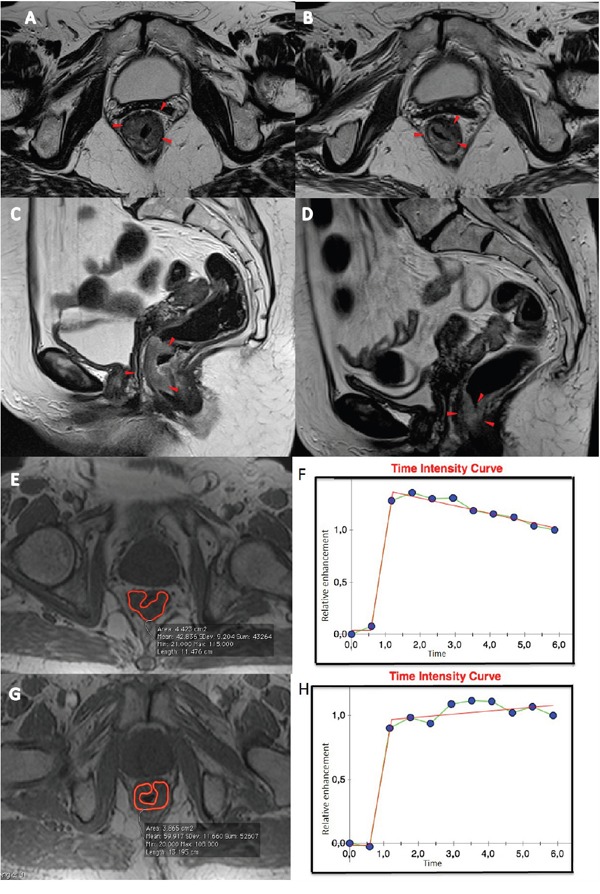
Patient n. 68: T2-weighted images in axial **A** and sagittal **C** plan before **B** the treatment and after treatment **D.** The morphologic images (A and C) before CRT, showed heterogeneous irregular thickening along the rectal wall spreading into the perirectal fat (A and C, arrowheads). After CRT, a hypointense area with thickening of the rectal wall and straining into perirectal fat (B and D, arrowheads) is observed. Median Time intensity curve of the volume of interest **E** and **G**, before **F** and after treatment **H** are shown. These curves showed different contrast enhancement, with a ΔSIS value of 39.54% classifying the patient as responder.

**Figure 5:**
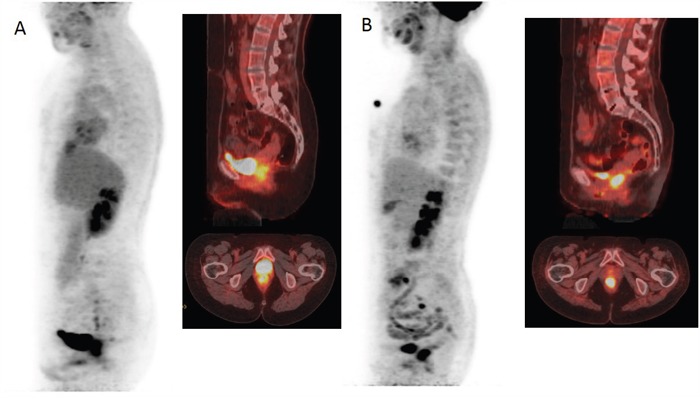
^18^F-FDG PET/CT images before **A** and after **B** treatment demonstrating a minimal reduction of glucose metabolism with ΔSUVmax of 6.07% classifying the patient as non responder.

Figure 1a shows ROC analysis for ΔSIS and ΔSUV_max_ in discriminating responders from non responders. The optimal cut-off for ΔSIS was a reduction of 6.0% yielding 92.7% of sensitivity and 80.0% of specificity to identify responder patients. Instead, the optimal cut-off of 59.7% for ΔSUV_max_ showed lower accuracy in identifying responder patients than ΔSIS, with a sensitivity of 67.3% and a specificity of 75.0%. 55 patients were classified as responders by ΔSIS, including 51 true positives, while 41 patients were classified as responders by ΔSUV_max_, including 37 true positives. The combination of ΔSIS and ΔSUV_max_ did not increase predictive ability, classifying 43 patients as responders, of whom only 36 were true pathological responders. Figure 1b shows ROC analysis for ΔSIS and ΔSUV_max_ in discrimination pathological complete response (TRG1) by incomplete response (TRG 2-4). The optimal cut-off for ΔSIS was a reduction of 30.3% (93.3% of sensitivity and 68.9% of specificity) while the optimal cut-off of 43.9% for ΔSUV_max_ showed lower accuracy (sensitivity of 80.0% and specificity of 31.1%). Statistically significant differences between ΔSIS and ΔSUV_max_, in terms of both sensitivity and specificity, were assessed using the McNemar test (p value <0.05), for both analysis. The presurgical PET/CT analysis demonstrated a low level of correlation between median ΔSUV_max_ value with pT and TRG findings (Spearman's rank correlation coefficient = -0.2 and -0.3, respectively), while a good level of correlation was observed between median ΔSIS value and pT and between median ΔSIS value and TRG (Spearman's rank correlation coefficient = -0.6 and -0.7, respectively). Table 3 shows the performance of ΔSIS and ΔSUV analysis to identify responder from non-responder patients and complete by incomplete pathological response. Figures 2 and 3 show DCE-MRI and ^18^F^-^FDG PET/CT images respectively, for a responder patient (TRG = 1). Figures 4 and 5 respectively show DCE-MRI and ^18^F^-^FDG PET/CT images, for another responder patient (TRG = 2)recognized only to ΔSIS analysis.

**Table 3:** Diagnostic Performance of ΔSIS and ΔSUV_max_

	Cut-off %	Sensitivity %	Specificity %	PPV %	NPV %	ACC %	AUC
*To differentiate responders (TRG1-2) by non responders (TRG 3-5)*							
*ΔSUV_max_*	59.7	67.3	75.0	88.1	45.5	69.7	0.71
*ΔSIS*	6.0	92.7	80.0	92.7	80.0	89.3	0.86
*To differentiate complete pathological response (TRG) by incomplete response (TRG 2-5)*							
*ΔSUV_max_*	43.9	80.0	31.1	43.6	70.0	50.7	0.57
*ΔSIS*	30.3	93.3	68.9	66.7	93.9	78.7	0.82

## DISCUSSION

The aim of the study was validate the potential of DCE-MRI (by means of ΔSIS value) in comparison to PET/CT (by means of ΔSUVmax) to evaluate preoperative neoadjuvant CRT response in LARC patients. There is a growing need to optimize the multidisciplinary management of patients with LARC, considering on the one hand that tumour response and patient benefit from CRT may considerably vary and on the other that preoperative treatment and TME are not completely free from serious early and late morbidity. In this scenario, the identification of patients with TRG 1–2, usually associated with a low prevalence of nodal involvement and a better outcome [26], would allow candidates to be selected for conservative mini-invasive strategies or for a “wait-and-see” policy [27–29].

Some authors reported the value of DCE-MRI based on semi-quantitative parameters such as initial slope, initial peak, late slope, and area under time intensity curve [30] or kinetic features (Ktrans, kep, ve) [31] in the evaluation of pathological complete response to pCRT in LARC. Martens et al. [30] concluded that “late slope” derived from DCE-MRI analysis using a semi-quantitative approach could predict before the beginning of pCRT which tumors are likely going to respond. Tong et al. [31] concluded that DCE-MRI could differentiate between pathological complete and incomplete pCRT response using a Ktrans threshold value of 0.66 reaching the 100% of sensitivity. Furthermore, some studies have shown how PET evaluation can predict pathologic tumor response and outcome after preoperative CRT in LARC patients, suggesting its great potential in assisting physicians on individualized management decisions in this disease [7–8, 10]. Several authors studied the benefit of apparent diffusion coefficient (ADC) of DWI and SUV of PET/CT in the assessment of pCRT response in LARC [32–34] showing that their combination allows to increase the sensitivity of the correct detection of response than either approach alone. However, a systematic review [34] reported a low positive predictive value (PPV) to predict pathological complete response (PPV of 54% and 39% for DWI and PET/CT, respectively). Baseline CRT imaging is not capable to forecast pathological complete response with overall accuracies of 68-72% for DWI and 44% for PET/CT. Qualitative DWI evaluation after CRT (5-10 weeks after the end) may outperform apparent diffusion coefficient reaching an overall accuracy of 87% versus 74-78%. The major strength of DWI and PET/CT is the capability to identify the non-responder patients who are not candidates for organ preservation. However, both DWI and PET/CT are not accurate enough to safely identify patients candidates for conservative mini-invasive treatments of for “wait and watch” policy allowing organ-sparing.

Our results show that ΔSUV_max_, between basal and pre-surgery SUV values, showed a significant correlation to TRG (AUC 0.71) with a sensitivity of 67.3%, a specificity of 75.0% and an accuracy of 69.7%, considering the optimal cut-off value of 59.7% provided by ROC analysis while a lower accuracy is shown to identify pathological complete response (sensitivity of 80.0% and specificity of 31.1%). Moreover, our results showed that ΔSUV_max_ median values were statistically different at Mann-Whitney U test for responder and not responder patients based on TRG. These findings with ^18^F-FDG PET/CT, using Standardized Uptake Value, are in agreement with previous results [10, 35–38]. Avallone et al. [10] reported that early changes of SUVmax were predictive of pathological response with an optimal threshold value of -42.0% and an accuracy of 93.0%. In this study, the authors also observed that the findings obtained from late PET scans, performed before surgery, showed lower accuracy in predicting pathologic response. Leccisotti et al. [35] evaluated metabolic modifications in the tumour during and after pCRT in 124 patients affected by LARC. A reduction of 61.2% of SUV was the best threshold to depict complete pathological response obtaining a 85.4% of sensitivity and a 65.2% of specificity while they [35] did not identify the optimal cut-off for the late response after PCRT. Leccisotti et al. [35] concluded that the PET/CT can predict early pCRT response depicting non-complete responders and allowing modification of treatment; contrariwise, late response before surgery is not sufficiently accurate for guiding the surgical decision versus TME, conservative strategies or observation over time. Niccoli-Asabella et al. [36] reported similar findings. Kim et al. [37] demonstrated that post-CRT SUVmax had a sensitivity of 60.4%, a specificity of 65.0%, and an accuracy of 55.9 %. Palma et al. [38] reported that post-CRT SUVmax had a sensitivity of 45.0%, a specificity of 70.0%, and an accuracy of 60.0%. Similar results were observed on advanced esophageal cancer [39]. Overall these data show the poor accuracy of late metabolic response to predict pathological responses, while they support the usefulness of performing PET/CT early during preoperative CRT in LARC.

Using ΔSIS analysis, we obtained better results than ΔSUV_max_, both in terms of sensitivity (92.7%), negative predictive value (92,7%) and accuracy (89.3%), considering the optimal threshold of 6.0%. These results are comparable with the findings reported in our previous paper [13] where ΔSIS percentage variation obtained a sensitivity of 93.5% and a specificity of 82.1%. ΔSIS showed a statistically significant difference in median values for responder and non-responder patients based on TRG and pathological T stage. In addition, a good linear correlation between ΔSIS median values and TRG score (Spearman's rank correlation coefficient = -0.7), was also observed.

Diagnostic performance of ΔSIS to assess preoperative CRT response was statistically significant in comparison of ΔSUV_max_ performance resulting an increase of sensitivity of 25.4% and an increase of negative predictive value of 34.5% (McNemar test p value <0.05). Moreover, an increase of ΔSIS diagnostic performance respect to ΔSUVmax was also observed in the differentiation of pathological complete response by incomplete response (ΔSIS cut-off of 30%): 13.3% of sensitivity increase, 37.8% of specificity increase, 23.1% of PPV increase and 23.9% of NPV increase. However, ^18^F-FDG PET/CT evaluation remains a more widely applicable approach to predict neo-adjuvant therapy response in LARC, whereas SIS is for the time being a promising DCE-MRI angiogenic biomarker with great potential for assessing preoperative treatment response and directing surgery for more or less conservative treatment.

The heterogeneity in the neoadjuvant treatment scheme with the majority of study population receiving an experimental schedule of “antiangiogenic” agent plus oxaliplatin in comparison of standard CRT scheme was previously investigated in the our study [13]. The analysis in [13] showed that the treatment schedule did not influence the proportions in responder and non-responder patients.

Some potential limitations deserve a special consideration: two radiologists assessed the MR images in agreement and in a single session per patient so that the intra-observer variability analysis was not performed. Butylscopolamine, dicyclomine, glucagon or similar drugs were not administered; however we performed volumetric analysis that minimize errors due to caused voxel misalignments.

Future improvement of this application could be the 1) development of an easy to use and user friendly SIS evaluation software, 2) comparison of SIS analysis with diffusion and perfusion coefficients obtained by Diffusion Weighted Imaging data analysis, 3) combination of multiple functional biomarkers (SIS, SUV, Diffusion Coefficients) to early predict neoadjuvant therapy response in LARC.

In conclusion, our study proposes an imaging angiogenetic biomarker, the Standardized Index of Shape, as an objective measurable index, easily transferable to clinical routine through a user-friendly software application, able to assess pCRT tumor response with a reproducible semi-quantitative measure of tumor blood perfusion. SIS percentage change could play an important role in LARC management helping to identify significant pathological response in order to adopt conservative strategies and to detect complete pathological response in order to guide versus a “wait and see” policy, reducing substantial morbidity and functional complications of TME.

## MATERIALS AND METHODS

### Patient selection

75 consecutive patients - with a median age of 62 years (range 44-77 years) were enrolled in this prospective study, from March 2007 to June 2014. All patients had a biopsy-proven rectal adenocarcinoma. Endorectal ultrasonography, pelvis MRI and whole body contrast enhanced CT scans were used for staging. Inclusion criteria were: patients with clinical T3-4 or with nodal involvement. Exclusion criteria were: inability to give informed consent, previous rectal surgery and contraindications for undergoing MRI or administering MR contrast media. Fifty-four (72%) patients had been enrolled in a phase II prospective trial previously described [9]. The study was approved by the Independent Ethical Committee of our institution. All patients gave written informed consent to participate to the study.

### Neoadjuvant therapy and surgical approach

External radiation therapy was performed using a 3-field technique (one posterior-anterior and two lateral fields). Standard fractions of 1.8 Gy/day to the reference point were given, 5 times a week up to a total dose of 45 Gy. Details of treatment planning have been previously reported [9]. Fifty-four patients received an experimental treatment with biweekly bevacizumab at 5 mg/kg plus three biweekly cycles of oxaliplatin at 100 mg/m^2^ and raltitrexed at 2.5 mg/m^2^ on day 1, and levo-folinic acid at 250 mg/m^2^, and 5-Fluorouracil at 800 mg/m^2^ on day 2 [8]. 21 remaining subjects received standard treatment with capecitabine at a dose of 825 mg/m^2^ twice daily, 5 days a week, for 5 weeks.

Patients underwent TME 8 (±1) weeks after completing CRT. An anterior or abdominoperineal resection was performed on the basis of the results of restaging.

### FDG-PET data acquisition and analysis

PET studies were acquired 60 min after the administration of 300–385 MBq of FDG either with a General Electric Discovery DST 600 PET/CT scanner [10]. All calibrations on the scanners to obtain accurate SUV readings were regularly performed. Patients fasted for at least 6 h, and blood glucose level was <150 mg/dl. Each patient underwent the baseline and the pre-operative study on the same scanner.

^18^F-FDG PET/CT image assessment was performed in a single reading session for each patient by consensus of two expert investigator with at least 15 years of experience. The readers were blinded to the clinicopathologic outcome and MRI findings. Irregular volumes of interest (VOIs) were semi-automatically drawn on orthogonal planes using a dedicated workstation and software using an arbitrary threshold, as reported previously [10]. For each patient both studies were analyzed at the same time in order to minimize discrepancies in VOI positioning. For each study maximum SUV (SUV_max_) values of the rectal lesion were recorded. FDG PET analysis results was performed by comparing measurements obtained in the rectal lesion at baseline (SUV_1_) and after treatment (SUV_2_). This change (known also as response index) was expressed as the percentage of SUV reduction (ΔSUV = (SUV_1_−SUV_2_)/SUV_1_×100) [9].

### MRI data acquisitions

All patients underwent DCE-MRI before and after CRT. Imaging was performed with a 1.5T scanner (Magnetom Symphony, Siemens Medical System, Erlangen, Germany) equipped with a phased-array body coil. Patients were placed in a supine, head-first position. Mild rectal lumen distension was achieved with 60-90 mL of undiluted ferumoxil (Lumirem, Guerbet, Roissy CdG Cedex, France) suspension introduced per rectum in order to obtain mild distension of rectal lumen [21] and improve the evaluation of rectal wall involvement, particularly in the post contrast MR scan. Pre-contrast coronal T1w 2D turbo spin-echo images and sagittal, coronal and axial T2w 2D turbo spin-echo images of the pelvis were obtained. Subsequently, axial, dynamic, contrast-enhanced T1w, FLASH 3D gradient-echo images were acquired for semi-quantitative MRI analysis. We obtained one sequence before and ten sequences, without any delay, after the IV injection of 0.2 mL/kg of a positive, gadolinium-based paramagnetic contrast medium (Gd-DOTA, Dotarem, Guerbet, Roissy CdG Cedex, France). The contrast medium was administered using a Spectris Solaris® EP MR (MEDRAD Inc., Indianola, PA) injector, with a flow rate of 2 mL/s, followed by a 10-mL saline flush at the same rate. Sagittal, axial and coronal post-contrast T1w 2D turbo spin-echo images, with and without fat saturation were obtained. The axial images were acquired without any angulation. Axial T1-w pre- and post-contrast sequences were acquired at the same position as the T2-w sequence. MRI total acquisition time was around 30 minutes. Sequence parameters details were reported in Table 4.

**Table 4:** Pulse Sequence Parameters on MR studies

Sequence	Orientation	TR/TE/FA (ms/ms/deg.)	AT (min)	FOV (mmxmm)	Matrix	ST/Gap (mm/mm)	TF
T1w 2D TSE	Coronal	499/13/150	2.36	450×450	256×230	3 / 0	3
T2w 2D TSE	Sagittal	4820/98/150	4.17	260×236	256×139	3 / 0	13
T2w 2D TSE	Coronal	4820/98/150	4.17	260×236	256×139	3 / 0	13
T2w 2D TSE	Axial	3970/98/150	3.48	270×236	256×157	3 / 0	13
T1w FLASH 3D	Axial	9.8/4.76/25	0.58	330×247	256×192	3 / 0	/
T1w FLASH 3D	Axial	9.8/4.76/25	0.58×10	330×247	256×192	3 / 0	/
T1w 2D TSE	Sagittal	538/13/150	2.35	250×250	256×230	3 / 0	5
T1w 2D TSE	Coronal	538/13/150	2.52	250×250	256×230	3 / 0	5
T1w 2D TSE	Axial	450/12/150	2.31	270×236	256×202	3 / 0	5

Abbreviations: TR = Repetition Time, TE = Echo Time, FOV = Field of View, FA = Flip Angle, ST = Slice Thickness, TF = Turbo Factor, AT = Acquisition Time

Spin-echo diffusion-weighted echo-planar imaging at different b values was performed for a limited subgroups of patients for this reason is not effected its analysis in this manuscript but could be object of a future study.

### MRI image data analysis

Image assessment was performed in a single reading session for each patient by consensus of two gastrointestinal radiologists with 13 years and 5 years of experience in reading pelvic MR images. MRI readers were blinded to the clinicopathologic outcome and PET/CT findings.

Regions of interest (ROIs) to cover the entire tumor volume were manually drawn slice by slice on pre-contrast T1-weighted images using the T2-weighted images as a guide [22]. Attention was placed to cover the entire lesion with the exclusion of peripheral fat, artefacts and blood vessels. Median values were recorded for all acquired tumor slices for each study.

For SIS analysis, for each TIC, the maximum signal difference (MSD) and wash out slope (WOS) were calculated, considering median value percentage change (ΔMSD=(MSD_1_ − MSD_2_)/MSD_1_×100 and ΔWOS= (WOS_1_ − WOS_2_)/WOS_1_×100), their linear combination (equation 1) was evaluated as described in our previously paper [13]:

Semi-quantitative analyses were carried out using Matlab R2007a software (The Math-Works Inc., Natick, MA).

### Evaluation of pathologic response

Details of how pathologic response assessment was performed have been described [8]. Briefly, surgical specimens containing the tumour were evaluated and scored according to tumour regression grade (TRG), as proposed by Mandard et al. [24] by two experienced pathologists who were not aware of MRI and FDG PET findings. Patients with a TRG 1 or 2 score were considered responders, whereas the remaining patients (TRG 3, 4, or 5) were classified as non responders.

### Statistical analysis

All quantitative data values were expressed as median ± standard deviation (SD) and compared with Mann-Whitney test. Chi-square test was performed to evaluate differences between pathologic responders (TRG 1-2) and non-responders (TRG 3-4) regarding baseline patient and tumour characteristics. Receiver operating characteristic (ROC) curves were calculated using ΔSIS and ΔSUV_max_ and optimal thresholds were obtained maximizing the Youden index. Sensitivity, specificity, positive and negative predictive value (PPV and NPV), for ΔSIS and ΔSUV, to differentiate responders by non responders patient and pathological complete response (TRG1) by incomplete response (TRG2-4) were performed. Matched sample tables and the McNemar Chi-square tests were used to compare the performance. Spearman's rank correlation coefficient was used to evaluate correlation between ΔSIS and ΔSUV_max_ with TRG and pathological T stage (pT).

P value <0.05 was considered significant for all tests. All analyses were performed using Statistics Toolbox of Matlab R2007a (The Math-Works Inc., Natick, MA).
